# First United States experience of robotic-assisted minimally invasive inguinal hernia repair using the Dexter surgical system

**DOI:** 10.1007/s00464-026-13223-4

**Published:** 2026-07-31

**Authors:** Nikita S. Thareja, Graham J. Spurzem, Ana Garcia Cabrera, J. Jeffery Reeves, Hannah M. Hollandsworth, Bryan J. Sandler, Garth R. Jacobsen, Ryan C. Broderick

**Affiliations:** Division of Minimally Invasive Surgery, University of California, San Diego (UCSD), 9300 Campus Point Drive, La Jolla, CA 92037 USA

**Keywords:** Dexter Surgical System, Novel robotic systems, Inguinal hernia repair, Learning curves, CUSUM

## Abstract

**Background:**

Robotic-assisted inguinal hernia repair continues to evolve with the introduction of novel surgical platforms designed to improve costs, workflow integration, and ergonomics. The Dexter Surgical System has recently entered clinical practice; however, its use for inguinal hernia repair in the USA has not been reported. This study evaluated feasibility, safety, and early operative outcomes in a United States cohort.

**Methods:**

A retrospective review was conducted of consecutive patients undergoing robotic inguinal hernia repair with Dexter at a single academic center between November 2024 and November 2025. Primary outcomes included intraoperative complications, device-related adverse events, and conversion to open or laparoscopic surgery. Secondary outcomes included operative time, console time, docking time, length of stay, and 30-day outcomes. Learning curve analysis was performed using cumulative sum methodology, along with a comparison of the primary surgeon’s first five versus final five cases.

**Results:**

Twenty-six patients underwent robotic inguinal hernia repair performed by two fellowship-trained minimally invasive surgeons with extensive prior robotics experience. All procedures were completed without conversion, complications, or device-related events, and all patients were discharged the same day. Mean operative time was 63.1 ± 18.5 min, with mean console time of 38.5 ± 15.7 min and docking time of 6.1 ± 2.2 min. One patient (3.8%) developed postoperative pneumonia requiring admission and intravenous antibiotics; no other 30-day complications, readmissions, or reoperations occurred. CUSUM analysis identified an inflection point at case six, after which operative times trended below the mean. Mean operative time decreased from 66.8 ± 11.1 min in the first five cases to 54.0 ± 13.3 min in the final five.

**Conclusion:**

This first United States experience demonstrates that robotic inguinal hernia repair using Dexter is feasible and safe in the outpatient setting, with favorable outcomes, efficient operative times, and a rapid learning curve among experienced surgeons.

Robotic-assisted surgery has become increasingly established in minimally invasive inguinal hernia repair, with platforms such as the Da Vinci Surgical System (Intuitive Surgical, Sunnyvale, CA) demonstrating comparable clinical outcomes, improved ergonomics, and enhanced dexterity compared with conventional laparoscopy [1–5]. However, the widespread adoption of robotic technology remains limited by system-related constraints, including operating room infrastructure requirements, docking complexity, and substantial capital and per-case costs [6–9]. These factors have driven the development of alternative robotic platforms aimed at improving accessibility, costs, and workflow efficiency [10, 11].

The Dexter Surgical System (Distalmotion, Geneva, Switzerland) is a novel modular robotic surgical platform designed to integrate into standard laparoscopic workflows while preserving the advantages of robotic-assisted minimally invasive surgery [12, 13]. The system utilizes conventional laparoscopic instrumentation and cameras, is compatible with standard electrical supply, and features an open console design that can be sterilely draped, allowing the surgeon to remain scrubbed and transition freely between console and bedside tasks [10, 12]. These design features are intended to reduce set-up complexity, improve workflow integration, and potentially facilitate adoption in ambulatory and high-throughput surgical settings [13, 14].

International studies have demonstrated the feasibility of the Dexter system across multiple general surgical procedures [12–17], and the system has received FDA de novo approval for use in inguinal hernia repair in the USA. However, clinical data for inguinal hernia repair in the USA have not been reported. Additionally, real-world assessments of operative efficiency, workflow integration, and early learning curve characteristics have not been conducted to determine whether the Dexter system’s theoretical design advantages translate into measurable clinical outcomes [13, 16, 17].

To address these gaps, we report the first clinical experience using the Dexter system for elective minimally invasive inguinal hernia repair in the USA. The objective of this study was to evaluate procedural feasibility, perioperative safety, and early operative efficiency, with additional assessment of the platform-specific learning curve in a cohort of experienced minimally invasive surgeons.

## Materials and methods

### Study design and setting

A retrospective case series was conducted at a single tertiary academic medical center, evaluating all consecutive patients undergoing elective inguinal hernia repair using the Dexter Surgical System between November 2024 and November 2025. Institutional review board approval was obtained before data collection.

### Surgeon experience

All procedures were performed by two fellowship-trained minimally invasive surgeons (R.C.B. and G.R.J.), who are both senior attending surgeons with over 7 years of experience in independent surgical practice. Both surgeons had extensive prior experience in advanced laparoscopic and robotic surgery, including routine use of the Da Vinci Surgical System for robotic inguinal hernia repairs, and prior participation in early adoption studies of novel robotic platforms.

### Patient selection

All patients undergoing elective minimally invasive inguinal hernia repair with the Dexter Surgical System during the study period were included consecutively. No formal exclusion criteria related to medical comorbidity, prior abdominal surgery, or hernia characteristics were established for the use of the platform. Procedures were performed at an ambulatory surgery center, where patient eligibility for outpatient surgery was determined by institutional and anesthesiology protocols. Therefore, the study cohort reflects patients deemed appropriate for outpatient inguinal hernia repair rather than a specifically selected Dexter-only population.

### Surgical technique

All cases were performed using a standardized transabdominal preperitoneal (TAPP) approach and laparoscopic port configuration [18]; minor modifications to port placement were made based on surgeon preference and patient factors. Following pneumoperitoneum and port placement, the robotic system was docked according to manufacturer-recommended setup protocols. The open console was positioned within the operating room for direct visual and verbal communication with the bedside assistants and sterilely draped for easy transitions between the console and bedside tasks. All operative steps, including docking and console phases, were standardized across cases. A ProGrip mesh was used in all cases, and all cases were performed with a Dexter-compatible needle driver, bipolar Maryland dissector, and bipolar Johann grasper.

### Data collection

Demographic variables, comorbidities, and operative characteristics were collected from the institutional electronic medical record. Perioperative variables included total operative time (incision to close), console time, docking time, conversion to open or laparoscopic surgery, intraoperative complications, and length of stay. Complications were defined as any unexpected intraoperative or postoperative event occurring within 30 days of surgery that required additional evaluation, treatment, readmission, or reoperation. Device-related adverse events were defined as any robotic system malfunction, technical issue, or patient injury attributable to the device that required troubleshooting, procedural modification, or conversion. 30-day postoperative outcomes were also captured, including complications, readmissions, and reoperations.

### Outcomes

The primary outcomes were operative feasibility and intraoperative safety, defined as successful completion of the procedure without conversion, intraoperative complications, or device-related adverse events. Secondary outcomes included operative times, length of stay, and 30-day postoperative outcomes.

### Learning curve analysis

CUSUM analysis was performed on 18 consecutive cases performed by the primary surgeon (R.C.B.) using the primary surgeon’s mean operative time (54.8 min) as the reference value. Sequential case numbers were plotted on the *x*-axis against cumulative deviation from the mean operative time on the *y*-axis; the CUSUM inflection point, defined as the case at which the curve transitions from a sustained upward to a sustained downward trend, was used to characterize the point of early platform performance stabilization [19]. The second surgeon’s case volume (*n* = 8) was insufficient to generate a meaningful independent learning curve analysis. A separate early versus late operative efficiency comparison was performed for the primary surgeon by comparing total operative time, console time, and docking time between the first five and final five cases.

### Statistical analysis

Descriptive statistics were used to summarize the data. Continuous variables are presented as mean ± standard deviation with ranges, while categorical variables are reported as counts and percentages. CUSUM analysis was performed based on deviation from the mean operative time to evaluate trends in operative performance over time.

## Results

### Patient and hernia characteristics

Twenty-six patients underwent inguinal hernia repair using the Dexter Surgical System during the study period. Patient demographics are summarized in Table 1. The mean age of the cohort was 59.8 ± 12.9 years, and the mean body mass index was 26.3 ± 3.6 kg/m^2^. The cohort was predominantly male (22/26, 84.6%) and White (16/26, 61.5%). The cohort encompassed a clinically relevant range of patient complexity; 3 patients (11.5%) were ASA class I, 16 (61.5%) were ASA class II, and 7 (26.9%) were ASA class III. Eight patients (30.8%) had prior abdominal surgery, including right hemicolectomy, laparoscopic sleeve gastrectomy with concurrent cesarean section and bilateral salpingo-oophorectomy, appendectomy, transanal minimally invasive rectal surgery, laparoscopic hiatal hernia repair, and nephrectomy. Four patients (15.4%) had undergone prior contralateral inguinal hernia repair, and no patient had prior ipsilateral inguinal hernia repairs.

**Table 1:** Patient demographics for the study cohort (*n* = 26)

Characteristic	Value, *n* (%) or mean ± SD (*n* = *26)*
Age, years (mean ± SD)	59.8 ± 12.9
Sex	
Male	22 (84.6%)
Female	3 (11.5%)
Non-binary	1 (3.8%)
BMI, kg/m^2^ (mean ± SD)	26.3 ± 3.6
Race	
White	16 (61.5%)
Multiracial	4 (15.4%)
Black	2 (7.7%)
Asian	2 (7.7%)
Middle Eastern	1 (3.8%)
Unknown	1 (3.8%)
Ethnicity	
Non-Hispanic	19 (73.1%)
Hispanic	6 (23.1%)
Unknown	1 (3.8%)
ASA physical status	
Class I	3 (11.5%)
Class II	16 (61.5%)
Class III	7 (26.9%)
Smoking status	
Never	15 (57.7%)
Former	7 (26.9%)
Current	4 (15.4%)
Prior abdominal surgery	8 (30.8%)
Prior contralateral hernia repair	4 (15.4%)

Values are presented as mean ± SD or *n* (%)

Prior hernia repairs were all contralateral; all index hernias in this series were primary

*BMI* body mass index, *ASA* American Society of Anesthesiologists

Hernia characteristics are summarized in Table 2. All 26 cases were primary inguinal hernias; the 4 patients with prior hernia repairs had undergone contralateral repairs. Hernia types included indirect hernias in 16 cases (61.5%), direct hernias in 8 cases (30.8%), a femoral hernia in 1 case (3.8%), and a combined indirect/direct hernia in 1 case (3.8%). Sixteen cases were right-sided (61.5%), and ten were left-sided (38.5%); no bilateral repairs were performed.

**Table 2:** Hernia characteristics for the study cohort (*n* = 26)

Characteristic	Value, *n* (%) (*n* = 26)
Hernia laterality	
Right	16 (61.5%)
Left	10 (38.5%)
Bilateral	0 (0%)
Hernia type	
Indirect	16 (61.5%)
Direct	8 (30.8%)
Femoral	1 (3.8%)
Mixed (indirect + direct)	1 (3.8%)
Hernia classification	
Primary	26 (100%)
Recurrent	0 (0%)

Values are presented as counts and percentages

### Operative and postoperative outcomes

**Fig. 1:**
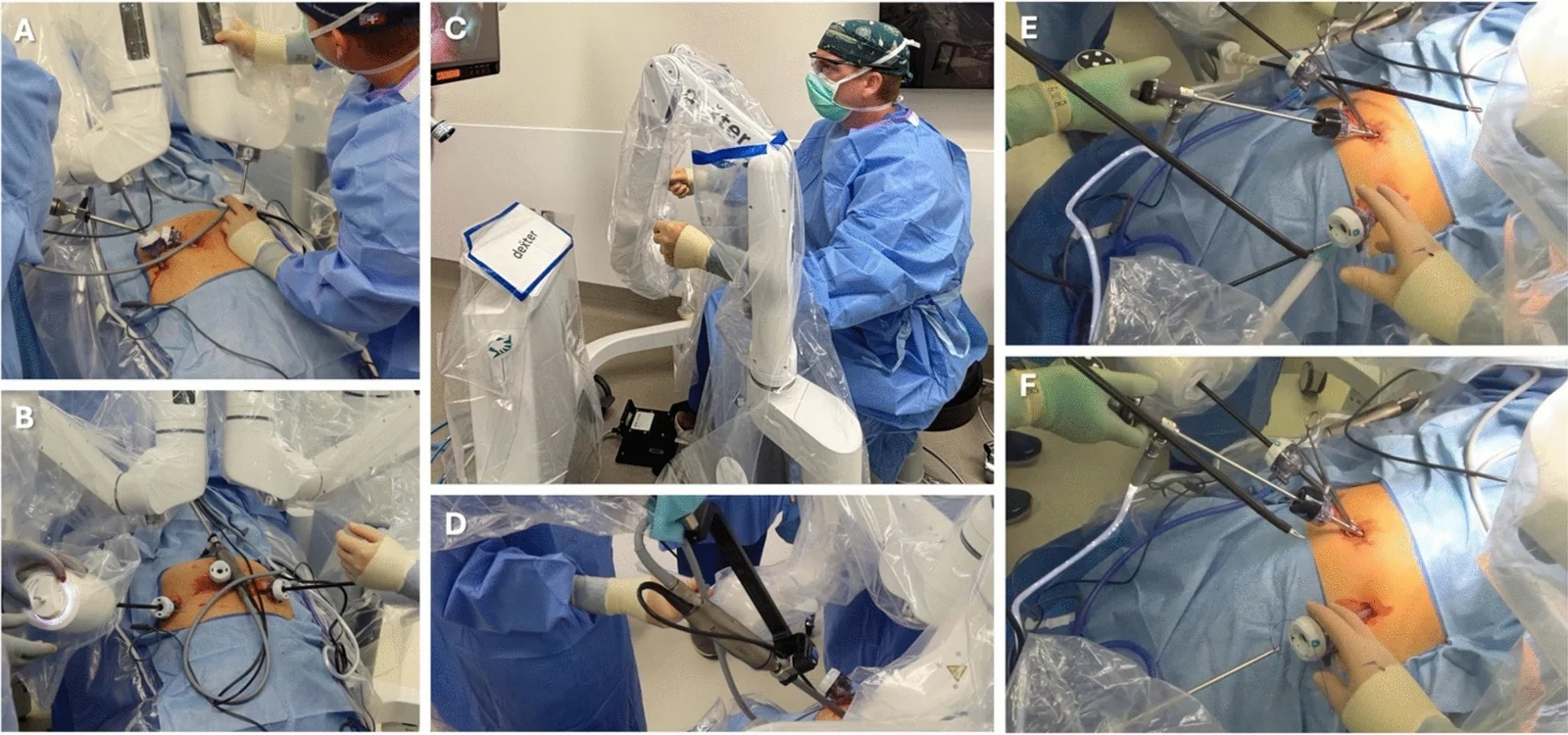
Representative intraoperative images of the Dexter Surgical System. **A** Docking the Dexter Surgical System. **B** Instrument and camera trocar positioning. **C** Operating surgeon at the sterilely draped open console. **D** Bedside assistant maintaining easy access to laparoscopic cameras. **E** Insertion of mesh into the abdomen. **F** Insertion of needles into abdomen

**Fig. 2:**
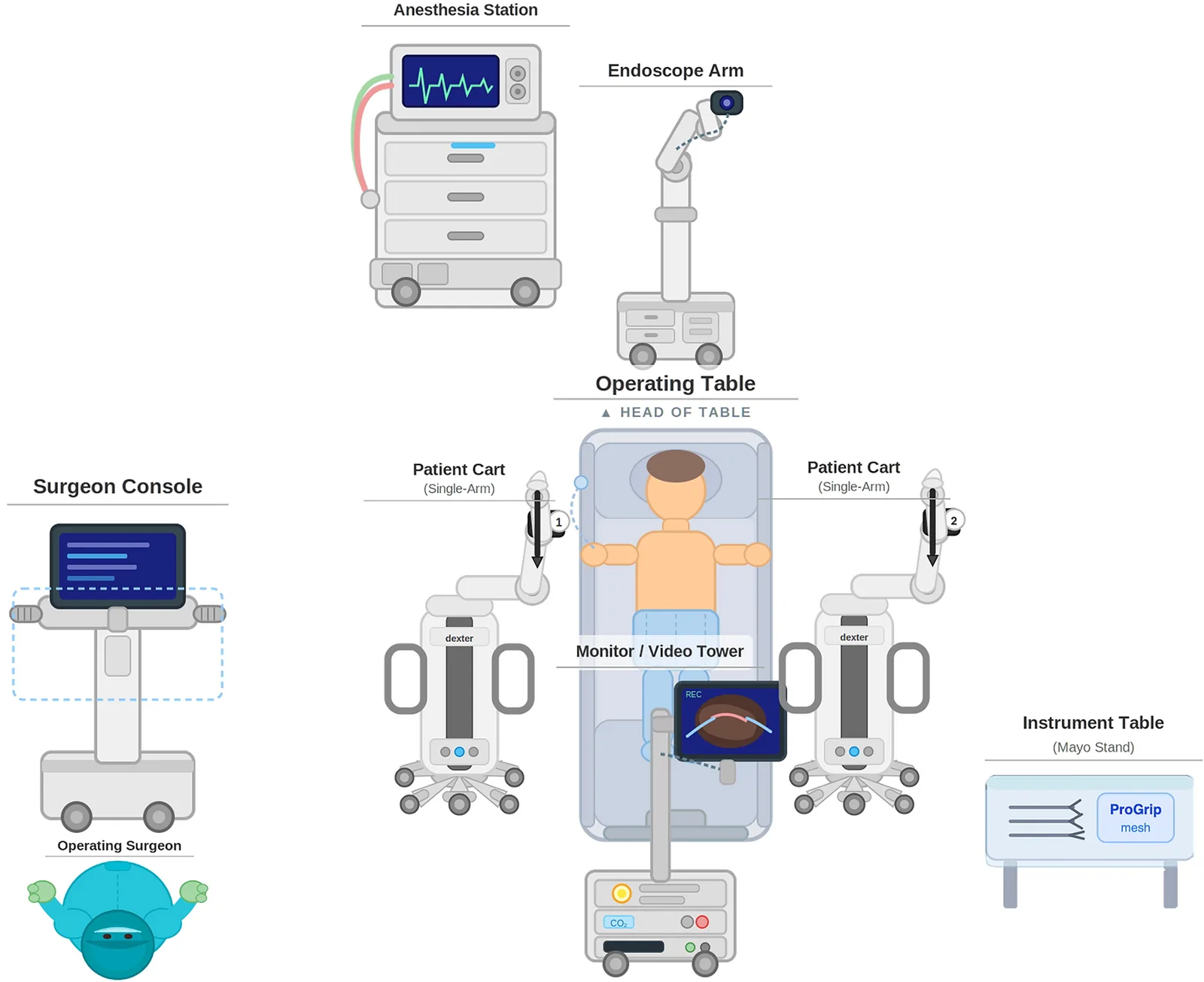
Schematic of operating room layout with modular three-cart Dexter system and open surgeon console. The image depicts locations of the surgeon console, operating table, patient arm carts, anesthesia carts, monitors, and instrument table

All procedures were completed successfully using the robotic platform without conversion to open or conventional laparoscopic surgery. There were no intraoperative complications or device-related adverse events. Preperitoneal ProGrip mesh placement was completed as planned in all cases. Intraoperative and postoperative outcomes are summarized in Table 3. Representative images of the Dexter system and operative setup are provided in Figs. 1 and 2, respectively.

**Table 3:** Intraoperative and 30-day postoperative outcomes (*n* = 26)

Outcome	Value, *n* (%) (*n* = 26)
Intraoperative outcomes	
Conversion to open surgery	0 (0%)
Conversion to conventional laparoscopy	0 (0%)
Intraoperative complications	0 (0%)
Device-related adverse events	0 (0%)
Postoperative outcomes	
Same-day discharge	26/26 (100%)
30-day complications	1 (3.8%)ᵃ
30-day readmission	1 (3.8%)ᵃ
30-day reoperation	0 (0%)

Values are presented as counts and percentages

ᵃOne patient was discharged on the day of surgery and readmitted ten days postoperatively for pneumonia requiring intravenous antibiotics. This event was deemed unrelated to the robotic platform

The mean total operative time was 63.1 ± 18.5 min (range 34–115 min), with a mean console time of 38.5 ± 15.7 min (range 21–96 min) and a mean docking time of 6.1 ± 2.2 min (range 2–12 min). Console times accounted for approximately 61% of total operative time, consistent with expected workflow distributions for robotic-assisted TAPP repair in which initial dissection, mesh deployment, and peritoneal closure are performed at the console. Operative times were comparable to institutional benchmarks for robotic inguinal hernia repairs (Table 4).

**Table 4:** Operative time metrics for the overall cohort (*n* = 26) and early versus late case comparison for the primary surgeon (R.C.B., *n* = 18)

Metric	Overall (*n* = 26)	Primary surgeon Early cases (*n* = 5)	Primary surgeon Late cases (*n* = 5)
Total operative time, min (mean ± SD)	63.1 ± 18.5	66.8 ± 11.1	54.0 ± 13.3
Console time, min (mean ± SD)	38.5 ± 15.7	41.8 ± 12.9	29.6 ± 4.9
Docking time, min (mean ± SD)	6.1 ± 2.2	7.0 ± 2.8	4.8 ± 1.1

Early vs. late comparison uses the first five (1–5) and final five (14–18) consecutive primary surgeon cases. The overall column includes all 26 cases from both surgeons

Primary surgeon is Dr. Ryan C. Broderick. Early vs. late comparison uses the first 5 (B1–B5) and final 5 (B14–B18) consecutive primary surgeon cases. The overall column includes all 26 cases from both surgeons

All patients were discharged home on the day of surgery, and there were no extended hospitalizations. One patient was admitted ten days postoperatively for pneumonia requiring intravenous antibiotics; this event was deemed unrelated to the robotic platform. There were no 30-day reoperations in this cohort (Table 3).

### Operative efficiency and learning curve analysis

**Fig. 3:**
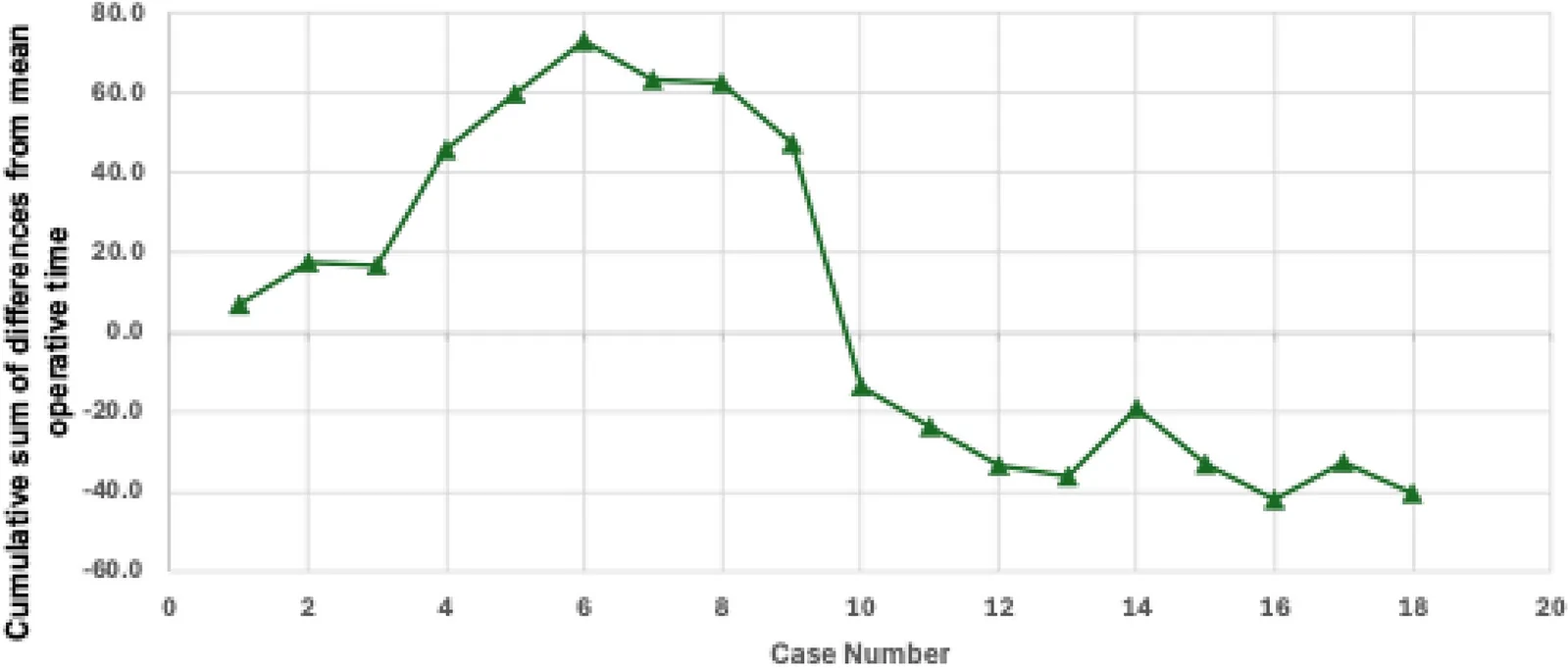
CUSUM learning curve analysis for the primary surgeon (R.C.B., *n* = 18 consecutive cases). The *x*-axis represents sequential primary surgeon case number (1–18); the *y*-axis represents the cumulative sum of deviation from the primary surgeon’s mean operative time (54.8 min). Positive values indicate cases above the mean; negative values indicate cases below the mean

CUSUM analysis of the primary surgeon’s 18 consecutive cases demonstrated a characteristic platform-specific learning pattern (Fig. 3). Using the primary surgeon’s mean operative time of 54.8 min as the reference, the CUSUM curve rose across the first six cases as operative times exceeded the surgeon’s mean, reaching a peak at the sixth primary surgeon case before transitioning to a sustained downward inflection. This inflection point is consistent with early platform performance stabilization. Given the surgeon’s extensive prior robotic experience, this adaptation likely reflects platform-specific skill acquisition—including familiarity with the Dexter console interface and instrument kinematics—rather than a general learning curve for robotic hernia repair.

A comparison of the first five versus the final five primary surgeon cases demonstrated progressive improvement across all operative time components (Table 4). Mean total operative time decreased from 66.8 ± 11.1 min to 54.0 ± 13.3 min (19% reduction). Mean console time decreased from 41.8 ± 12.9 min to 29.6 ± 4.9 min (29% reduction), while mean docking time decreased from 7.0 ± 2.8 min to 4.8 ± 1.1 min (31% reduction). These improvements in operative times suggest that efficiency gains were driven by improvements in console and docking efficiency rather than changes in room setup alone.

## Discussion

This study presents the first clinical experience in the USA using the Dexter Surgical System for inguinal hernia repair. In this early single-center series of 26 consecutive cases, all procedures were completed without conversion to open or conventional laparoscopic surgery, intraoperative complications, or device-related adverse events. All patients were discharged the same day, with one readmission for postoperative pneumonia deemed unrelated to the platform. These findings support the technical feasibility and perioperative safety of the Dexter system for outpatient minimally invasive hernia repair.

The safety profile observed is consistent with published benchmarks for robotic TAPP inguinal hernia repair [5, 20]. The 100% same-day discharge rate confirms that the system is suitable for outpatient settings, which is an important consideration given the increasing proportion of inguinal hernia repairs performed in ambulatory and free-standing surgical facilities [21, 22]. This cohort included 7 ASA class III patients and 8 patients with prior abdominal surgery, representing a clinically relevant range of patient complexity. However, this cohort only consisted of primary hernia repairs, and the system has not been evaluated in U.S. patients with recurrent inguinal hernias. Further studies are needed to assess performance in more complex patients and procedures.

Operative efficiency metrics, including total operative, console, and docking times, were comparable to institutional benchmarks for robotic TAPP repairs. The mean operative time of 63.1 min falls within ranges previously reported for robotic TAPP inguinal hernia repairs by experienced robotic surgeons [5, 23]. Console time accounted for approximately 61% of total operative time, consistent with the expected distribution for robotic TAPP repair in which initial dissection, flap creation, mesh positioning, and peritoneal closure are performed at the console [15, 24]. These findings suggest that integration of the Dexter system into an experienced minimally invasive surgical practice can be achieved without compromising operative performance during early adoption. The mean docking time of 6.1 min is particularly noteworthy, since comparative data for the Da Vinci system report experienced high-volume centers achieving docking times of approximately 5 min after workflow standardization [25]. This consistency of docking times, even in early cases, suggests that the system’s modular design and laparoscopic port compatibility facilitate efficient setup without a prolonged platform-specific docking learning curve. This may have practical implications for operating room efficiency, particularly in high-volume ambulatory surgical centers where setup and operative times limit scheduling capacity [26, 27].

Early versus late case comparisons demonstrated improvements across all operative components, with the greatest reductions in console (29%) and docking (31%) times, suggesting that efficiency gains were driven by increasing familiarity with console operation and docking workflow rather than operating room setup alone. CUSUM analysis identified a learning curve inflection point at case six, after which operative times consistently trended below the mean. Given the surgeon’s extensive prior robotic experience, this likely reflects platform-specific skill acquisition, consistent with prior literature demonstrating that novel systems require adaptation even among experienced users due to differences in instrument kinematics, console interface, haptic feedback, and system responsiveness between platforms [28, 29]. Given that prior robotic experience has been shown to facilitate more rapid adaptation [28, 30, 31], this has important implementation implications, as experienced surgeons may achieve early performance stabilization within relatively few cases if their robotic skills from other platforms are transferable. However, the high level of prior robotic experience in this cohort may limit generalizability, and less experienced surgeons or centers may require additional cases to achieve similar proficiency [30, 31]. Future prospective multicenter studies across diverse practice settings and experience levels are needed to further evaluate adoption patterns, learning curves, and reproducibility.

This rapid acclimation to the system may be partially explained by several of its design features, which could have implications for implementation and resource utilization. Its modular architecture and compatibility with standard laparoscopic equipment, power supply, and imaging systems appeared to simplify operating room setup during these initial cases, potentially reducing costs and the need for extensive retrofitting compared to larger integrated platforms [11, 32]. In addition, the open console design allows the surgeon to remain sterile while seated upright and maintaining direct visual and verbal communication with the operative team [11, 15], which may improve ergonomics, communication, and workflow flexibility compared to closed-console systems [33–36]. Although not formally measured, surgeons and trainees anecdotally noted potential ergonomic and communication advantages with this setup, which warrants future validation using standardized assessment tools. Collectively, these simplified setup and infrastructure requirements may facilitate broader adoption, particularly in ambulatory surgical centers where efficiency and turnover directly impact capacity [26, 27]. However, formal cost-per-case analyses and prospective comparative studies are needed to determine whether these perceived advantages translate into measurable improvements in operative efficiency and resource utilization [8, 37].

This study has several limitations that should be considered when interpreting these findings. Its retrospective design, small sample size, and single-center setting limit our ability to draw definitive conclusions regarding the system’s comparative effectiveness relative to established robotic platforms or its generalizability to other surgical practices [38]. Since all procedures were performed in an ambulatory surgery center, the cohort was inherently limited to patients considered suitable for outpatient surgery, which may limit the generalizability of these findings to higher-risk patients or those requiring inpatient postoperative care. The learning curve analysis was restricted to the primary surgeon’s cases due to insufficient case volume for the second surgeon, which further limits the generalizability of our CUSUM findings. Follow-up was also limited to 30 days, which precludes assessment of long-term outcomes such as hernia recurrence [39].

Despite these limitations, this study provides early clinical evidence supporting the feasibility and safety of this novel robotic platform for outpatient inguinal hernia repair in the USA and establishes a foundation for future comparisons between robotic platforms. Prospective studies will incorporate standardized workflow metrics, validated ergonomic assessment tools, and cost analyses to better define the system’s indications and its role within minimally invasive hernia surgery.

## Conclusion

The Dexter Surgical System is a feasible and safe platform for robotic-assisted inguinal hernia repair. In this first U.S. case series of 26 consecutive patients, all procedures were completed without intraoperative complications or device-related adverse events, and with operative times comparable to institutional benchmarks for established robotic systems. CUSUM learning curve analysis identified an inflection point at the primary surgeon’s sixth case, which suggests a rapid platform-specific adaptation among experienced robotic surgeons. The platform’s open console design and standard laparoscopic equipment compatibility represent design features with potential ergonomic and workflow advantages that warrant formal prospective evaluation.
